# Integrated traditional Chinese medicine for childhood asthma in Taiwan: a Nationwide cohort study

**DOI:** 10.1186/1472-6882-14-389

**Published:** 2014-10-10

**Authors:** Yu-Chiang Hung, I-Ling Hung, Mao-Feng Sun, Chih-Hsin Muo, Bei-Yu Wu, Ying-Jung Tseng, Wen-Long Hu

**Affiliations:** Department of Chinese Medicine, Kaohsiung Chang Gung Memorial Hospital and School of Traditional Chinese Medicine, Chang Gung University College of Medicine, Kaohsiung, Taiwan; School of Chinese Medicine for Post Baccalaureate, I-Shou University, Kaohsiung, Taiwan; China Medical University College of Chinese Medicine, Taichung, Taiwan; Institute of Public Health, China Medical University College of Public Health, Taichung, Taiwan; Management Office for Health Data, China Medical University Hospital, Taichung, Taiwan; Kaohsiung Medical University College of Medicine, Kaohsiung, Taiwan; Fooyin University College of Nursing, Kaohsiung, Taiwan

**Keywords:** Traditional Chinese medicine, Asthma, Emergency, Hospitalization

## Abstract

**Background:**

Traditional Chinese medicine (TCM) is the most commonly used alternative therapy in children with asthma, especially in the Chinese community. This study aimed to investigate the effects of the government-sponsored Outpatient’s Healthcare Quality Improvement (OHQI) project with integrated TCM treatment on childhood asthma.

**Methods:**

This study used the Longitudinal Health Insurance Database 2000, which is a part of the Taiwan National Health Insurance Research Database (NHIRD). Children with diagnosed asthma and aged under 15 years from 2006–2010 were enrolled. They were collated into 3 groups: (1) subjects treated with non-TCM; (2) subjects treated with single TCM; and (3) subjects treated with integrative OHQI TCM. The medical visits and the cost of treatment paid by the Bureau of National Health Insurance (BNHI) to the outpatient, emergency room, and inpatient departments were evaluated for the study subjects within 1 year of the first asthma diagnosis during the study period.

**Results:**

Fifteen multi-hospitals, including 7 medical centers, and 35 TCM physicians participated in OHQI during the study period. A total of 12850 children from the NHIRD database were enrolled in this study, and divided as follows: 12435 children in non-TCM group, 406 children in single TCM group, and 9 children in integrative OHQI TCM group. Although the total medical cost paid by the BNHI per patient in the integrative OHQI TCM group was greater than that in the non-OHQI groups, the patients in the integrative OHQI TCM group exhibited greater therapeutic effects, and did not require ER visits or hospitalization. In addition, ER visits and hospitalization among patients who received a combination of conventional therapy with integrated TCM were lower than those among patients who underwent conventional therapy alone or single TCM treatment.

**Conclusions:**

Asthmatic children at partly controlled level under conventional therapy may benefit from adjuvant treatment with integrated TCM.

## Background

Childhood asthma is a major allergic and chronic respiratory disease, and its prevalence has increased worldwide in recent years [1–6]. According to one report, 66.0% of asthmatic children in China had acute exacerbations, 26.8% received emergency treatment, and 16.2% were hospitalized [7]. Asthma exacerbation and wheezing resulted in more than 2 million incidences of school absenteeism and visits to the emergency room per year [8]. The medical cost paid by the Bureau of National Health Insurance (BNHI) for the utilization of emergency services, hospitalization, and outpatient physician care for asthmatic children was approximately two-fold higher than that for non-asthmatic children [9]. The caregiver burden on the patients’ families and government has increased to unprecedented levels [3, 10–12]. Specific public health interventions were conducted [13, 14] to reduce the higher rates of asthma hospitalization and use of associated medication among children [15].

Current evidence clearly supports the conventional approach of using inhaled bronchodilators and steroids as first-line agents. Unfortunately, the failure rate of conventional therapies has increased. In patients who fail to respond to initial treatment or are difficult to treat [16], complementary and alternative therapies may be of benefit [17–22]. Traditional Chinese medicine (TCM) is the most commonly used alternative therapy, especially in the Chinese community. Researchers have investigated various forms of TCM to treat asthma [23, 24], including Chinese herbal medicine [25–28], acupuncture ([29–31], massage [32], and herbal paste [33–35]. However, some results are controversial, suggesting the need for further research [36–39]. Therefore, this study investigated the impact of the government-sponsored Outpatient’s Healthcare Quality Improvement (OHQI) project with integrated TCM treatment on the frequency of emergency treatment or hospitalization for childhood asthma.

## Methods

This study was approved by the Intuitional Review Board in China Medial University Hospital (Permit No CMU-REC-101-012), Taiwan. The Taiwan National Health Insurance (NHI) program was a signal-payer system set up on March 1, 1995, by the BNHI. The BNHI-entrusted National Health Research Institutes constructed several National Health Insurance Research Databases (NHIRD) as part of this program. This study used the Longitudinal Health Insurance Database 2000 (LHID2000), which was a part of the NHIRD. The LHID2000 included information of 1 million individuals randomly selected from the 2000 Registry of Beneficiaries. This database contained information on all medical claims from 1996 to 2010, and disease was defined according to the International Classification of Diseases, 9^th^ Revision, Clinical Modification (ICD-9-CM).

**Figure 1:**
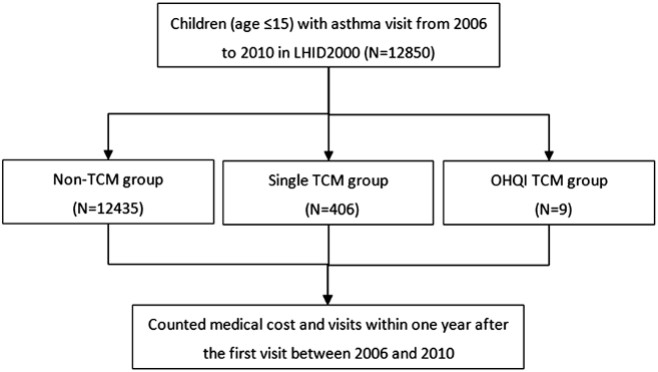
**Flow chart of subject recruitment from the Longitudinal Health Insurance Database 2000 (LHID2000) from 2006 to 2010 in Taiwan.** OHQI: Outpatient’s Healthcare Quality Improvement, TCM: traditional Chinese medicine.

Asthma is a common chronic inflammatory disease of the airways characterized by wheezing, coughing, chest tightness, and shortness of breath, reversible airflow obstruction, and bronchospasm. Children younger than 15 years in 2006–2010 who were diagnosed with asthma (ICD-9-CM 493) by pediatric specialists were selected, because the OHQI project with integrated TCM for childhood asthma at the state of “partly controlled” level according to the guidelines from the Global Initiative for Asthma (GINA_Pocket_2014_Jun11) started in 2006. They were divided into 3 groups: (1) non-TCM: children treated with conventional therapy but without TCM; (2) single TCM: children treated with single TCM and conventional therapy; and (3) integrative OHQI TCM: children treated with integrated TCM using the application of OHQI and conventional therapy during the study period (Figure 1). The integrative OHQI TCM for childhood asthma involved application of Chinese herbal medicine, acupuncture, massage, and herbal paste (Table 1). According to the patients’ syndrome (Zheng) differentiation, the representative prescriptions such as Xiao-Qing-Long-Tang would be prescribed for phlegm-cold pattern or Ding-Chuan-Tang for phlegm-heat pattern.

**Table 1:** **The Outpatient**’**s Healthcare Quality Improvement with integrated traditional Chinese medicine for childhood asthma**

Intervention	Prescription
Chinese herbal medicine	*Xiao*-*Qing*-*Long*-*Tang* or *Ding*-*Chuan*-*Tang*
Acupuncture	*YuJi* (LU10), *Zusanli* (ST36)
Massage	Governor Vessel and Bladder Meridian on the back
Herbal paste	*Feishu* (BL13), *Fengmen* (BL12), *Dazhui* (GV14)

Ingredients of *Xiao*-*Qing*-*Long*-*Tang*: *Ephedrae Herba*, *Cinnamomi Ramulus*, *Paeoniae Radix*, *Zingiberis Rhizoma*, *Pinelliae Tuber*, *Asiasari Radix*, *Schisandrae Fructus*, *Glycyrrhizae Radix*.

Ingredients of *Ding*-*Chuan*-*Tang*: *Ephedrae Herba*, *Mori Radicis Cortex*, *Armeniacae Semen*, *Glycyrrhizae Radix*, *Perillae Fructus*, *Scutellariae Radix*, *Pinelliae Tuber*, *Tussilaginis Flos*.

The medical visit rate, visit frequency, and cost paid by the BNHI on the behalf of the study subjects within 1 year after the first date of asthma diagnosis during the study period were determined. The distribution of gender and medical visits included outpatient (OPD), emergency (ER), and inpatient (IPD) visit data, and a chi-squared test or Fisher’s exact test was used if the cell count was <5. The Wilcoxon two-sample test and Kruskal-Wallis test were used to compare the median frequency of visits and cost paid by the BNHI between the groups. Dunn’s test was used for post-hoc analysis among the three groups. All analyses were performed using SAS statistical software (version 9.3 for Windows; SAS Institute, Inc., Cary, NC, USA) and the significance level was set at *p* <0.05.

## Results

Fifteen multi-hospitals (including 7 medical centers) and 35 TCM physicians participated in the OHQI during 2006–2010. In order to investigate the differences in outcome between childhood asthma with OHQI and without OHQI, data for 12850 children were selected from the NHIRD, including that for 12435 children treated without TCM, 406 children treated with single TCM, and 9 children treated with OHQI integrated with TCM. There were more girls in the integrative OHQI TCM group, but more boys in the other 2 groups. We found a male predominance among asthmatic children [total male/female: 7707(60%)/5143(40%)] and the TCM (single TCM or integrative OHOI TCM) therapy groups [male/female: 255(61.45%)/160(38.55%)] (Table 2). The lowest median age was 9.29 years in the non-TCM group, followed by 10.1 years in the single TCM group and 11.2 years in the integrative OHQI TCM group. Most of the asthmatic children lived in urban areas. Within 1 year after the first asthma visit in 2006–2010, the median cost for OPD and the total cost paid by the BNHI were highest in children that received treatment with integrative OHQI TCM, followed by children that received single TCM and conventional therapy without TCM. However, asthmatic children that received integrative OHQI TCM neither visited the ER nor were they hospitalized during the study period.

**Table 2:** **Medical visits and cost paid by the BNHI for study subjects from the NHIRD**

	Non-TCM		Single TCM		OHQI		
	N = 12435		N = 406		N = 9		p-value
Sex, n (%)							0.47
Girl	4983	(40.1)	155	(38.2)	5	(55.6)	
Boy	7452	(59.9)	251	(61.8)	4	(44.4)	
Age, median (SD)	9.29	(4.04)	10.1	(4.14)	11.2	(2.51)	<0.0001^3(a)^
Urbanization							0.25
Urban	7397	(59.5)	258	(63.5)	5	(55.6)	
Rural	5038	(40.5)	148	(36.5)	4	(40.5)	
All OPD, n (%)	12121	(97.5)	406	(100.0)	9	(100.0)	0.005
Visit, median (IQR)	3	(5)	6	(13)	6	(13)	<0.0001^3(a,c)^
Cost, median (IQR)	1642	(4177)	3777	(9202)	27180	(16380)	<0.0001^3(a,b,c)^
OPD for TCM							
Visit, median (IQR)	--		3	(4.5)	6	(7)	0.31^2^
Cost, median (IQR)	--		1545	(3315)	17600	(17920)	0.0001^2^
ER, n (%)	1244	(10.0)	60	(14.8)	0	(0.00)	0.007^1^
Visit, median (IQR)	1	(1)	1.5	(1)	--		0.001^2^
Cost, median (IQR)	1179	(1443)	1956	(2589)	--		0.002^2^
IPD, n (%)	124	(1.00)	9	(2.22)	0	(0.00)	0.09^1^
Visit, median (IQR)	1	(0)	1	(1)	--		0.07^2^
Cost, median (IQR)	9675	(7165)	12013	(11066)	--		0.37^2^
Total medical cost, median (IQR)	12496	(4271)	17746	(10162)	27180	(16380)	<0.0001^3(a,b,c)^

Chi-squared test, ^1^Fisher’s exact test, ^2^Wilcoxon two-sample test and ^3^Kruskal-Wallis test. ^a^Non-TCM vs. OHQI TCM was significant, ^b^Single TCM vs. OHQI TCM was significant, ^c^Non-TCM vs. Single TCM was significant. *BNHI*: Bureau of National Health Insurance, *NHIRD*: National Health Insurance Research Database, *OHQI*: Outpatient’s Healthcare Quality Improvement, *TCM*: Traditional Chinese Medicine. *IQR*: Interquartile range.

Asthmatic children that received single TCM treatment had a higher ER visit rate (14.8%) than children that did not receive TCM treatment (10%), but the ER or IPD visit rate did not differ significantly between these 2 groups (Table 2 and Table 3). The median costs paid by the BNHI for emergency services and hospitalization were New Taiwan Dollar (NTD) 1956 and 12013, respectively, in asthmatic children that received conventional therapy with single TCM, and were higher than the NTD 1179 and 9675 respectively observed in those that received only conventional therapy without TCM. In addition, the former’s medical cost paid by the BNHI was NTD 3777, being the sum of NTD 1545 for single TCM and NTD 2232 for conventional therapy, which was more expensive than the latter’s costs of NTD 1642 (Table 2). However, the ER or IPD medical costs were not significantly different between the conventional therapy without TCM group and the treatment with single TCM group (Table 2 and Table 4).

**Table 3:** **Medical visits by the BNHI for study subjects from the NHIRD in age**-**adjusted linear regression**

	Mean	SD	Estimation (95%)	P
All OPD				
Non-TCM (N = 12435)	3.61	2.82	Reference	
Single TCM (N = 406)	7.00	7.09	3.56 (3.11, 4.00)	<0.0001
OHQI TCM (N = 9)	5.38	3.62	2.14 (−0.61, 4.90)	0.13
OPD for TCM				
Non-TCM (N = 12435)	0			
Single TCM (N = 406)	5.12	5.54	Reference	
OHQI TCM (N = 9)	5.71	3.50	0.71 (−3.44, 4.87)	0.74
ER				
Non-TCM (N = 12435)	1.39	0.82	Reference	
Single TCM (N = 406)	1.50	0.82	0.12 (−0.18, 0.43)	0.44
OHQI TCM (N = 9)	0			
IPD				
Non-TCM (N = 12435)	1.17	0.54	Reference	
Single TCM (N = 406)	1.33	0.50	0.16 (−0.20, 0.53)	0.38
OHQI TCM (N = 9)	0			

*BNHI*: Bureau of National Health Insurance, *NHIRD*: National Health Insurance Research Database, *OHQI*: Outpatient’s Healthcare Quality Improvement, *TCM*: Traditional Chinese Medicine.

**Table 4:** **Medical cost paid by the BNHI for study subjects from the NHIRD in age**-**adjusted linear regression**

	Mean	SD	Estimation (95%)	P
All OPD				
Non-TCM (N = 12435)	2847.2	4288.6	Reference	
Single TCM (N = 406)	5661.1	7745.2	2944.8 (2446.7, 3442.8)	<0.0001
OHQI TCM (N = 9)	16898.3	12017.3	14351 (11240, 17462)	<0.0001
OPD for TCM				
Non-TCM (N = 12435)	0			
Single TCM (N = 406)	3966.4	6711.6	Reference	
OHQI TCM (N = 9)	19004.3	10996.8	15338 (10193, 20483)	<0.0001
ER				
Non-TCM (N = 12435)	1573.6	1253.2	Reference	
Single TCM (N = 406)	1881.3	1228	316.6 (−153.1, 786.3)	0.19
OHQI TCM (N = 9)	0			
IPD				
Non-TCM (N = 12435)	11996.9	11073.5	Reference	
Single TCM (N = 406)	13374.1	7440.4	1314.6 (−611.8, 8740.1)	0.73
OHQI TCM (N = 9)	0			
Total medical cost, median (IQR)				
Non-TCM (N = 12435)	3087.8	4976.6	Reference	
Single TCM (N = 406)	6182.9	8680.8	3229.5 (2655.2, 3803.8)	<0.0001
OHQI TCM (N = 9)	16898.3	12017.3	14122 (10528, 17715)	<0.0001

*BNHI*: Bureau of National Health Insurance, *NHIRD*: National Health Insurance Research Database, *OHQI*: Outpatient’s Healthcare Quality Improvement, *TCM*: Traditional Chinese Medicine.

## Discussion

The total medical cost paid by the BNHI in the integrative OHQI TCM group was greater than that of the non-OHQI groups. However, asthmatic children in the integrative OHQI TCM group did not visit the ER or undergo hospitalization during the study period. In addition, combined treatment with TCM could reduce school absenteeism due to hospitalization or ER visits, and restore the normal working lives of the patient’s parents. Therefore, the caregiver burden on patients’ families and the government resulting from childhood asthma would decrease. Moreover, parents who do not require leave to care for their children would continue working, resulting in increased national productivity. The main objectives of integrative OHQI TCM for asthmatic children sponsored by the government were to (1) alleviate the severity of asthma, (2) reduce the frequency of the utilization of emergency services and hospitalization, and (3) decrease the caring burden on patients’ parents. Our results showed that government-sponsored integrative OHQI TCM may have a substantial impact, and may achieve these goals.

Asthmatic children who received conventional therapy with single TCM had a higher ER visit rate and higher medical cost paid by the BNHI for emergency services and hospitalization than those for patients who received only conventional therapy without TCM. The conditions may be more severe in the former than in the latter, resulting from resistance to conventional therapy. However, the integrative OHQI TCM therapy had a greater impact with regard to improving the ER or IPD care of asthmatic children than single TCM interventions. Consistent with a previous report, our findings showed better results with combined therapy than with monotherapy [40].

*Xiao*-*Qing*-*Long*-*Tang* is indicated in asthma with whitish sputum and nocturnal cough, while *Ding*-*Chuan*-*Tang* is indicated in asthma with yellowish sputum. *Xiao*-*Qing*-*Long*-*Tang* may attenuate allergic airway inflammation [41], and prevent asthma through neurotropin regulation [42]. *Ding*-*Chuan*-*Tang* may improve airway hyper-responsiveness in stabilized asthmatic children [43]. Acupuncture at *YuJi* (LU10) and *Zusanli* (ST36) could regulate cardiopulmonary function, Fas and Bcl-2 mRNA expression, and promote eosinophil apoptosis in the asthmatic state [44, 45]. Massage at the Governor Vessel and Bladder Meridian on the back can improve key pulmonary functions in asthmatic children, namely, FEV_1_ and the FEV_1_/FVC ratio [32]. Applying a herbal paste (ingredients: *Sinapis alba L*. seeds, *Asarum heterotropoides Fr. Schmidt var. mandshuricum* (*Maxim*.) *Kitag*, *Euphorbia kansui T. N. Liou ex T. P. Wang*, *Corydalis yanhusuo W. T. Wang*, *Dryobalanops aromatica Gaertn. f*. and *Zingiber officinale Rosc*. juice) at *Feishu* (BL13), *Fengmen* (BL12) and *Dazhui* (GV14) may reverse the Th1/Th2 imbalance that is characteristic of asthma, through regulation of cell factors and their specific transcription factors, and may have a beneficial effect on asthma [46]. In summary, integrative OHQI TCM can moderate the immune state, and relieve inflammatory reactions in asthmatic children.

The Taiwan NHIRD showed that the number of asthmatic children in this population-based prospective study was increasing each year. Patients who fail to respond to conventional therapy may benefit from TCM. Therefore, the increasing prevalence of asthma warrants studies investigating the management of childhood asthma with integrated TCM. The number of childhood asthma patients that received OHQI increased from 239 in 2006 to 393 in 2010. This result reflects a consistent trend of an increasing prevalence of childhood asthma.

The limitation of this study was that only 9 asthmatic children that received integrative OHQI TCM were selected from the NHIRD. It is very likely that the different male/female ratio of the OHQI group is because of the limited number of cases. Since these 9 patients were randomly sampled from the one million patients, the results of this study maybe representative, but the outcome of decreased utilization of ER and hospitalization service could not be overstated. The other limitation was that a selection bias would exist in the study cohort. The LHID2000 includes data on a closed cohort since 2000 and does not include information of children younger than 6 years in 2006. In addition, making a definite diagnosis of asthma in children 5 years and younger is challenging because episodic respiratory symptoms such as wheezing cough are also common in children who do not have asthma, particularly in those younger than 3 years (GINA_Under5_Pocket_20091).

## Conclusions

There seemed to be a better therapeutic effect on integrative OHQI group in this study, their ER visits and hospitalizations were also decreased. Our findings suggest that asthmatic children at partly controlled level under conventional therapy may benefit from adjuvant treatment with integrated TCM.
